# Efficacy of the Rabbit Polyclonal Anti-leptospira Antibody against Homotype or Heterotype Leptospira Infection in Hamster

**DOI:** 10.1371/journal.pntd.0005191

**Published:** 2016-12-27

**Authors:** Xuemin Jin, Wenlong Zhang, Zhuang Ding, Hai Wang, Dianjun Wu, Xufeng Xie, Tao Lin, Yunhe Fu, Naisheng Zhang, Yongguo Cao

**Affiliations:** 1 Department of Clinical Science, College of Veterinary Medicine, Jilin University, Changchun, People's Republic of China, China; 2 Department of Infectious Disease, College of Veterinary Medicine, Jilin University, Changchun, People’s Republic of China, China; 3 College of Animal Science and Veterinary Medicine, Heilongjiang Bayi Agricultural University, Daqing, People's Republic of China, China; 4 Department of Chemistry and Biochemistry, South Dakota State University, Brookings, SD, United States; 5 Key Laboratory for Zoonosis Research, College of Veterinary Medicine, Jilin University, Changchun, People's Republic of China, China; Fondation Raoul Follereau, FRANCE

## Abstract

Leptospirosis, caused by *Leptospira*, is one of the most important of neglected emerging zoonotic diseases that has important impacts on public health worldwide. Polyclonal antibody (pcAb) therapy is a potential method to process a series of pathogens for which there are limited determination of treatment, such as leptospirosis. First, we evaluated the efficacy of pcAb, derived from the sera of rabbits inoculated with Leptospira, against homotype (*Leptospira interrogans* serovar Lai) or heterotype (*Leptospira interrogans* serovar Autumnalis) Leptospira infection in a lethal hamster model. The pcAb treatment improved survival compared to the controls. The histopathology’s of the infected kidney, liver and lung were also examined by hematoxylin and eosin staining. Using real-time quantitative PCR, we determined that most of the leptospires in the primary organs were almost completely removed by pcAb. In the second experiment, treatments, including antibiotic, pcAb, and combination, were started immediately after occurrence of the first serious sickness mouse in any group. No significant difference in survival rate between pcAb group and antibiotic group was found, but the combination therapy group significantly improved survival rate compared to the others (P<0.05). We conclude that the rabbit pcAb treatment may cure both the homotype and the heterotype lethal Leptospira infections in hamster, and combination therapy improved survival compared to antibiotic group in the late treatment of homotype leptospirosis.

## Author Summary

Leptospirosis, caused by *Leptospira*, is one of the most important neglected emerging zoonotic diseases that has important impacts on public health worldwide. An urgent need exists for novel therapeutic agents directed against Leptospira without severe side-effects. This study demonstrates the efficacy of a rabbit polyclonal antibody (pcAb) measured by survival rate, pathological changes and Leptospira burden. The pcAb can cure the homologous serotype and heterologous serotype Leptospira lethal infection 48 hours after challenge. We also provide a reference dose of pcAb for treating human or dog leptospirosis. In addition, combination of pcAb with antibiotic improved survival compared to antibiotic group in late treatment of homotype leptospirosis in hamster. However, additional clinical research is needed to identify the efficacy in humans.

## Introduction

Leptospirosis, caused by *Leptospira*, is one of the most important neglected emerging zoonotic diseases has great impacts on public health worldwide. Onset of Leptospirosis is in diverse epidemiological settings and more common in vulnerable populations living in poor conditions, such as rural residents and urban slum people [1–6]. Leptospirosis is one of the heading zoonotic causes of morbidity globally, and mortality rate is observed [7]. In many areas, leptospirosis is considered a major contributor to pulmonary hemorrhage and acute kidney injury [8–11].

Currently, antibiotic therapy is the most commonly used treatment against leptospirosis. However, antibiotics residue and the probability of multidrug resistance are concerns, which may influence the safety of patients while using the antibiotic therapy. For example, James E. Moon and Zhang W reported that nearly all animals exhibited diarrhea after some types of antibiotic therapies [12,13]. Therefore, an urgent need exists for novel therapeutic agents directed against Leptospira without severe side-effects.

In 1890, von Behring found that the immune rabbit sera were able to protect infected mice from diphtheria or tetanus. By 1894, anti-diphtheria serum was used in humans in Europe [14]. Phisalex and Bertrand proved that the blood of animals immunized with a European viper had antitoxin effects [15]. Serum therapy was also implemented in clinical use for bacterial infections including pneumococcal infection, meningococcal disease and streptococcal infection (scarlet fever) [16,17]. Due to sickness and anaphylaxis of the serum, antibiotics against bacterial infections were widely used in the 1940s, which replaced the use of serum therapy [16]. Subsequently, however, enzymatic digestion and purification techniques were rapidly developed, providing safer polyclonal antibody (pcAb) therapies for many diseases, such as envenomation, rabies exposure, varicella–zoster virus infection, respiratory syncytial virus and hepatitis A and B [17]. Currently, some animal studies demonstrated the efficacy of pcAb treatment for curing a variety of neglected tropical viral diseases. Passive immunization of Nipah virus glycoproteins protected hamsters against a lethal challenge by Nipah virus [18]. Polyclonal IgG given 48h after Ebola virus challenge provides complete protection in non-human primate [19]. And other studies of various animals including mice, monkeys and guinea pigs showed that purified immunoglobulin G (IgG) from horses prolongs survival period against Ebola virus [20–25].

Polyclonal serum therapy is a potential method to process a series of pathogens for which there are limited determination of treatment, such as leptospirosis. In 1986, S. Faine reported that administration of a monoclonal antibody from Balb/c mice before and after challenge with virulent homologous leptospires passively prevented lethal leptospirosis in new born guinea pigs [26]. It was reported that a rabbit polyclonal antibody, given before challenging with leptospires, protects hamsters against the homologous serotype Leptospira infection [27]. However, few studies regarding protection against the heterologous serotype Leptospira infection were performed, and in these few studies, treatment was initiated prior to challenge. We propose that pcAb can protect against the homologous serotype Leptospira infection, but doubt the efficacy of pcAb against the heterologous serotype Leptospira infection. By comparing the survival rate, histological features and bacterial load between the control group and experimental group, we examined the effect of polyclonal anti-Leptospira antibody against leptospirosis in a hamster model. In addition, in the second experiment, we tried to start treatment immediately after occurrence of the first serious sickness mouse in any groups and compare the effects between antibiotic, pcAb and combination against homotype Leptospira infection.

## Materials and methods

### Ethics statement

All animals were maintained on standard rodent chow with water supplied ad libitum with a 12/12h light/dark cycle during experimental period. All animal experiments followed the regulations for the Administration of Affairs Concerning Experimental Animals in China. The protocol was approved by the Committee on the Ethics of Animal Experiments of the First Norman Bethune Hospital of Jilin University, China [(2013) clinical trial (2013–121)].

### Leptospires

*Leptospira interrogans* serovar Lai (56601) and *Leptospira interrogans* serovar Autumnalis (56606), two significant pathogenic Leptospira in China, were from Dr. Xiaokui Guo. In all experiment, the low passage cultures were obtained from hamster tissue infected by leptospires.

### Animal models

Leptospirosis models in hamsters were established as previously reported [13]. Briefly, female golden Syrian hamsters (*Mesocricetus auratus*), 3–4 weeks of age and weighing 40–60 g were obtained from the Animal Center of Jilin University. The modified absolute lethal dose (MLD100) was obtained by challenging groups of 4 hamsters with 10-fold serial dilutions containing 10^7^ to 10^1^ leptospires in 1 ml volume via intraperitoneal injection, and the result showed that the MLD100 of 56601 and 56606 were approximately 10^7^ and 10^6^ organisms, respectively. The 7-day mortality rate of was 100% after challenge with leptospira. All golden Syrian hamsters were challenged by intraperitoneal injection with 0.5 ml of EMJH (Ellinghausen-McCullough-Johnson- Harris) liquid medium containing 10^7^ 56601 or 10^6^ 56606, which were determined using a Petroff–Hausser counting chamber under dark-field microscope.

### Rabbit immunization

As previously described [28], each rabbit was injected intravenously into the ear vein with a dose of well-grown 56601 containing approximately 2–4×10^8^ leptospires/ml according to the following schedule: day 1, 1 ml; day 6, 2 ml; day 11, 4 ml; and days 16 and 21, 6 ml each. One week after the last injection, antisera were collected by centrifuging clotted blood at 3,000 g. Normal sera were collected from normal rabbits.

### Microscopic agglutination test (MAT)

The antisera titer was determined by MAT [28] as follows: well-grown 56601 was used as an antigen. The serum was heated at 56°C for 30 min to inactivate the complement. Serial two-fold dilutions of the sera, starting with a dilution of 1:25, were mixed with an equal volume of 56601 in a microtiter plate. After incubation at 30°C for 2 hours, the antiserum was performed for agglutination using dark field microscopy. Final titers represent the reciprocal of the highest serum dilution showing at least 50% agglutination of antigen in the suspension [29].

### Purification and characterization of antibody

The IgG of pcAb (IgG-pcAb) and normal antibody were purified using the caprylic acid ammonium sulfate precipitation method of McKinney and Parkinson from the antisera and normal sera, respectively [30]. The concentration of the IgG-pcAb and normal antibody were determined with the BCATM Protein Assay Kit (Thermo, USA) and adjusted to a final concentration of 20 mg/ml with phosphate buffered saline (PBS). The antibodies were stored at -20°C until used for treatment. The titer of IgG-pcAb was determined by ELISA using microtiter plates that had been previously coated with leptospires (10^7^ leptospires/well). The antibody titers were defined as the highest dilution with an absorbance value (OD450) of more than twice blank control.

### Therapeutic trials

In the first experiment, IgG-pcAb were diluted with normal saline to different necessary concentrations, such as 8, 4, 2, 1, 0.5 and 0.25 mg/ml, for the following experiment. Hamsters, infected with 56601, were randomly divided into 7 groups (n = 8/group). Five groups were treated with IgG-pcAb at 16, 8, 4, 2, 1 and 0.5 mg/kg each, whereas the remaining 2 groups were the control groups, including a normal saline group and a normal antibody group. Hamsters in normal antibody group were administered normal antibody at 8 mg/kg. Subsequently, to evaluate the efficacy of the IgG-pcAb against *Leptospira interrogans* serovar Autumnalis (56606) infection, we randomly divided the hamsters (infected with 56606) into 3 groups (n = 8/group). Hamsters in the pcAb group were treated with 8 and 16 mg/kg of IgG-pcAb. Hamsters were subjected to subcutaneous injection once daily from day 3 to day 7 after challenge.

In the second experiment, hamsters infected with 56601 were started treatment immediately after occurrence of the first serious sickness mouse (appeared moribund) in any groups and compare the effects between antibiotic (n = 10/group), pcAb (n = 10/ group, 16 mg/kg via subcutaneous injection) and combination (antibiotic + pcAb, n = 10/group) against homotype Leptospira infection. Doxycycline at 5mg/kg (via intraperitoneal injection) of weight was used in antibiotic therapy. The dose administration of the remaining control groups (normal serum group and normal saline group) was as described above. Hamsters were administrated once daily for 5 days.

Remarkably, all hamsters were administrated a similar volume of drug, approximately 100 μl. After challenge with leptospires, all hamsters were observed no less than 3 times per day for a period of 21 days, during which serious sickness mouse appeared moribund was observed and then was humanely euthanized by CO_2_. The number of dead hamsters was recorded. The primary organs (liver, kidney, and lung) of the dead hamsters were collected. On day 21, the surviving hamsters were humanely euthanized by CO_2_, and the primary organs (liver, kidney, and lung) were also collected for the following experiments. The protective efficacy after challenge was determined in two independent experiments.

### Histology

The kidney, liver and lung of the surviving hamsters infected 56601 or 56606 from the pcAb group (8mg/kg) and dead hamsters from the control groups were fixed with 4% paraformaldehyde for 24 hours at room temperature and then embedded in paraffin and sectioned at a thickness of 4 μm. Pathological changes of organ slices were examined by hematoxylin and eosin (H&E) staining, and the organ injury index (the injured area/total organ area x100%) was calculated for each slice. The severity of leptospire induced lesions was graded as previous description [31].

### Real-time quantitative PCR assay

To measure the leptospiral load, leptospires from primary organs (kidney, liver and lung) were quantitatively measuring by quantitative PCR (qPCR). Eight control samples were randomly selected from control groups, and samples of pcAb group were from all hamsters treated by 8mg/kg pcAb. As described previously [13], tissue samples (0.09–0.15 g) from the organs were homogenized in 1 ml of sterile normal saline on ice and then centrifuged (1300 g, 10 minutes). Then DNA was extracted from the supernatants, after which the DNA concentration was determined by spectrometry. The qPCR assays were performed using previously published primers, optimized reaction mixtures and cycling parameters [32]. Establishing a standard curve analyzed by serial dilutions (10^9^–10^2)^ of DNA contributed to calculate the number of bacteria in the organ. Bacterial load was expressed as the amount of genome equivalents per μg kidney, liver and lung DNA [33].

### Statistical analysis

Kaplan–Meier plots for all groups were used to calculate the survival rate. Comparison of survival time between groups was analyzed using the log-rank test. P values <0.05 were considered significant. The average tissue scores for each group of hamsters were calculated by Tukey-Kramer pairwise analysis and P<0.05 was considered statistically.

## Results

### IgG-pcAb recognizes leptospires with high titer

**Fig 1:**
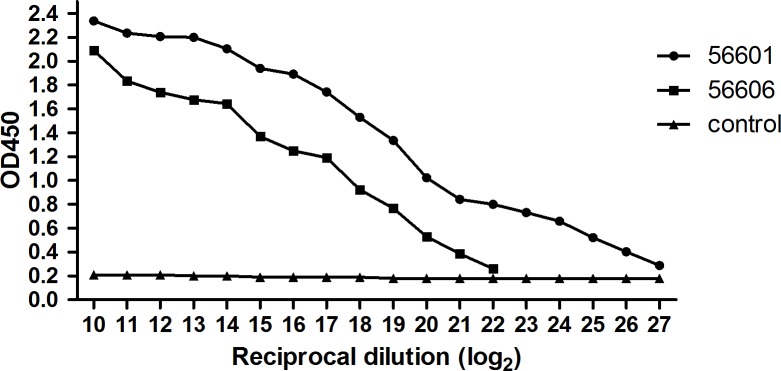
ELISA was used to determine the titer of IgG-pcAb. In brief, 96-well plates were pre-coated with 56601 or 56606. The concentration of purified rabbit-raised antibodies was adjusted to 20 mg/ml and then added to the well by a two-fold serial dilution (2^10^ to 2^27^). The OD450 was then determined. Data were presented as the mean from two separate experiments, each individually conducted in triplicate.

As shown in the Table 1, the titer of antisera against the live homologous serotype Leptospira (56601) was 3200 by MAT while normal serum did not occur agglutination (Table 1). IgG-pcAb interacted with 56601 or 56606 with high titer, ranging from 2^26^ and 2^21^ (Fig 1). Obviously, this IgG-pcAb was also able to react with 56606, a heterologous serotype leptospira. These results suggested that IgG-pcAb generated in this study was able to recognize 56601 and 56606 *in vitro*.

**Table 1:** Leptospiral agglutinating activity of antisera

Sera	Final agglutination titer								
	50	100	200	400	800	1600	3200	6400	12800
Antisera	+	+	+	+	+	+	+	-	-
Normal sera	-	-	-	-	-	-	-	-	-

### IgG-pcAb plays a positive role in survival

**Fig 2:**
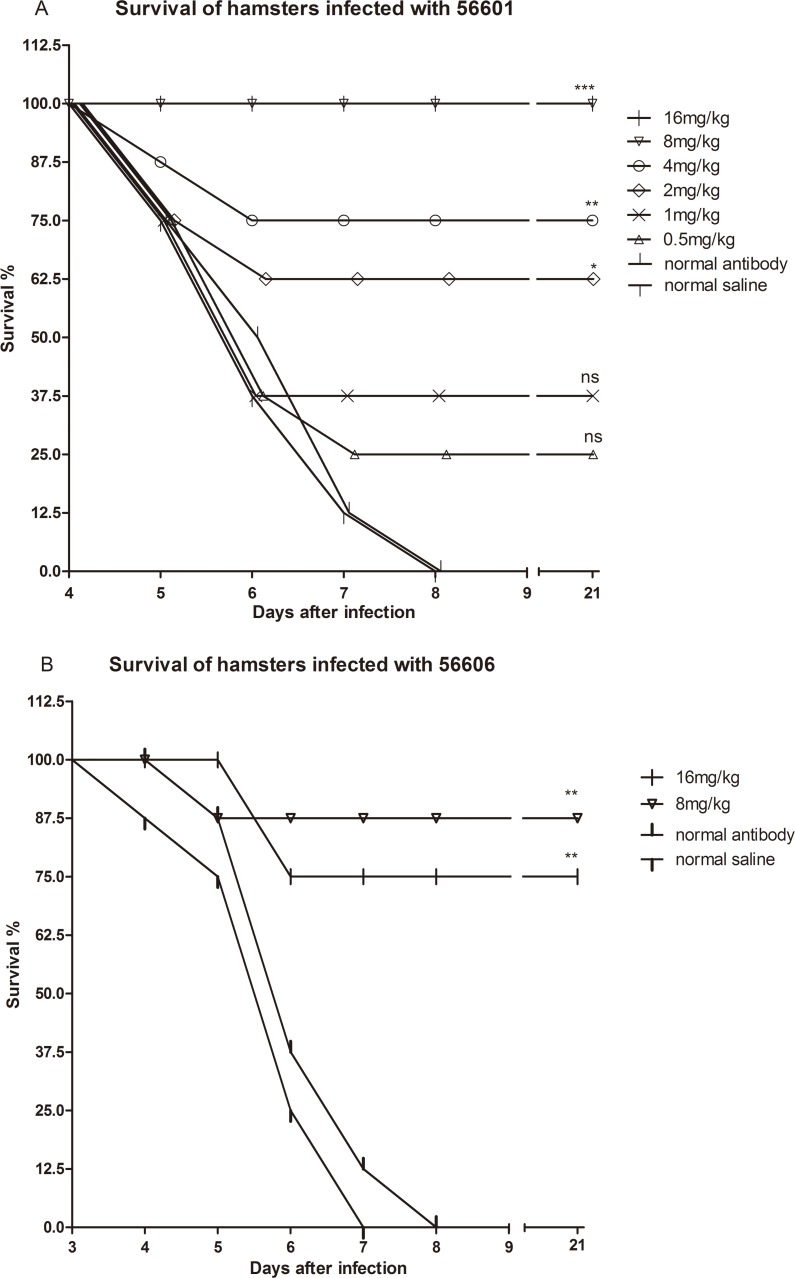
Survival of hamsters with acute leptospirosis treated with IgG-pcAb. (A) Efficacy of IgG-pcAb (16, 8, 4, 2, 1and 0.5 mg/kg) for treating acute homologous (56601) leptospirosis. (B) Efficacy of IgG-pcAb (16 and 8 mg/kg) for treating acute heterologous (56606) leptospirosis. Survival differences between study groups were compared using the log-rank test. *P<0.05, **P<0.01 and ***P<0.001 vs. the control group.

In the first experiment, after challenge with 56601, hamsters in the two control groups all died between day 5 to day 8. There was no significant difference between the normal saline group and normal antibody group. Therefore, the normal rabbit antibody did not interfere with survival based on the current evidence. Additionally, 16, 8, 4 and 2 mg/kg of IgG-pcAb significantly improved survival compared to controls, but the other 2 pcAb groups (1 and 0.5mg/kg) did not show improved survival. In particular, survivals of the16 and 8 mg/kg groups were 100% (Fig 2A). Consequently, pcAb treatment improved survival in a dose-dependent manner. As shown in Fig 2B, the normal saline group as not significantly different from the normal antibody group. Notably, the first serious sickness mouse occurred in the normal saline group on day 4 after challenge with 56606. However, pcAb treatment was also significantly efficient for improving the survival of the hamsters infected with 56606, but there were also limited lethality during heterologous leptpspiral infection. In summary, pcAb treatment improved survival compared to controls, but may not protect hamsters from heterologous infection completely.

**Fig 3:**
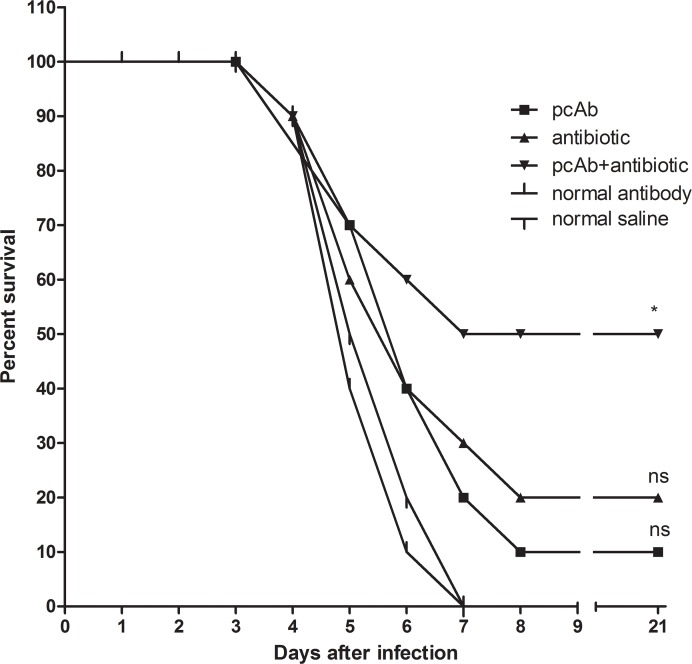
Survival of hamsters treated in the late leptospirosis. Efficacy of IgG-pcAb (16 mg/kg), antibiotic (doxycycline, 5mg/kg) and combination (pcAb plus antibiotic) were evaluated in treating acute and late homologous (56601) leptospirosis. Survival differences between study groups were compared using the log-rank test. *P<0.05 vs. the control group.

In the second experiment (Fig 3), survival curves of hamsters receiving pcAb or antibiotic were not significantly different. Survival rates of pcAb and antibiotic group were 10% and 20%, respectively, but half of hamsters treated with combination survived at the end of 21 days. Combination therapy significantly improved survival compared to the two control groups (P<0.05), but pcAb and antibiotic did not. The results showed that survival rates in hamsters receiving the combination therapy were higher than those receiving antibiotic or pcAb alone.

### Histopathological changes

**Fig 4:**
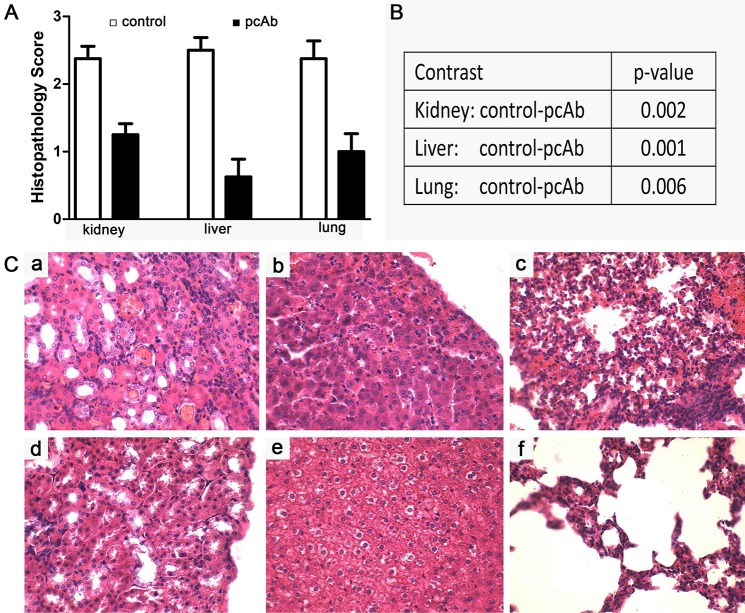
Histopathology and scores of kidney, liver and lung after infection with *Leptospira interrogans* serogroup Lai (56601) or Autumnalis (56606). (A) Histopathology scores of the kidney, liver and lung in hamsters. The data represent the mean histopathology scores for the two groups of hamsters. Statistical analysis of results for infected controls (n = 8) and pcAb group (n = 8) was performed using the Wilcoxon rank sum test. *p < 0.05. (B) Tukey-Kramer pair-wise comparisons with the control. (C) Histopathology of the kidney (a, d), liver (b, d), and lung (c, f) of infected controls (a, b, c) and the pcAb group (d, e, f) after infection with leptospira in hamsters. These representative photographs were selected from hamsters infected 56601 or 56606.

Representative photographs are from survived hamsters in pcAb group (8mg/kg), and from dead hamsters in control groups, regardless of 56601 infection or 56606 infection. Kidney, liver and lung lesion grades were lower in the pcAb group than in the infected controls (Fig 4A). There is significant difference between control group and pcAb group in histopathological scores (Fig 4B). Hemorrhages appear in renal tissues of control hamsters, but not in kidneys of the pcAb (8 mg/kg) treatment group (Figs 4Ca and 3Cd). Tight junction defects, areas of necrosis and inflammatory infiltration were observed in the livers of control hamsters. There were hepatic tight junctions and decreased necrosis in livers of hamsters treated with IgG-pcAb (Figs 4Cb and 3Cd). Hemorrhages were also observed from pulmonary tissues of controls. Moreover, we observed interstitial pneumonia with hyperemia of alveolar and mononuclear cell infiltration. However, there was little evidence of interstitial pneumonia and hemorrhages in lungs of surviving hamsters from the pcAb (8 mg/kg) group (Figs 4Cc and 3Cf).

### IgG-pcAb reduces leptospires burden of organs

**Fig 5:**
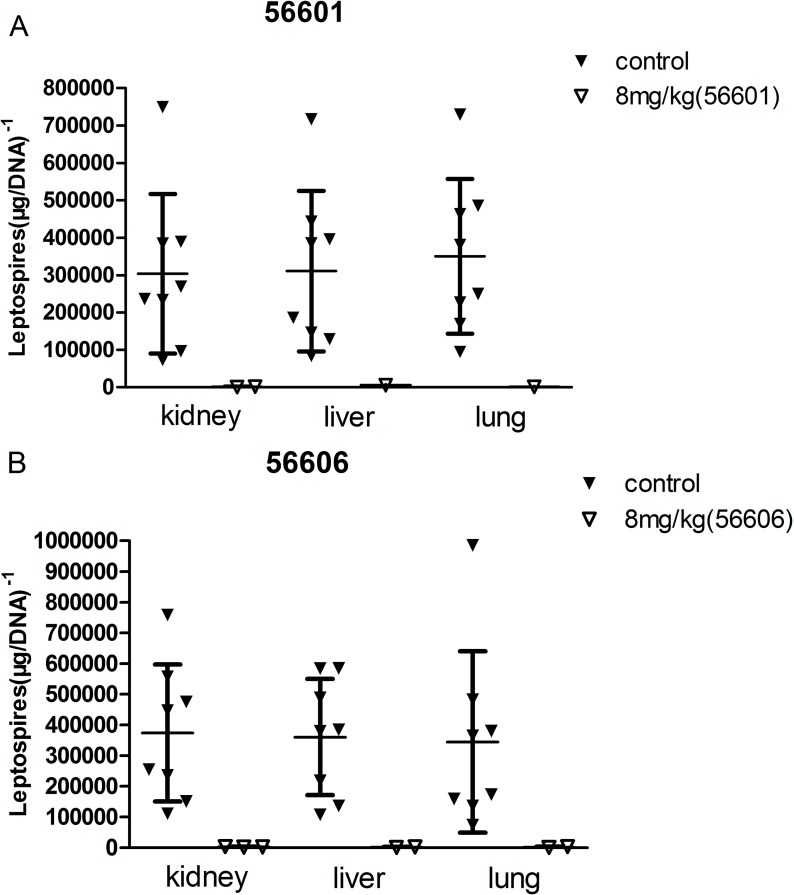
**56601 (A) and 56606 (B) burden of primary organs (kidney, liver and lung) as determined by qPCR.** The results are presented as the number of genome equivalents per μg kidney, liver and lung DNA with a scatter dot plot. Each dot represents a positive sample of organs. The results were presented as the mean from two separate experiments, each individually conducted in triplicate.

Eight samples from control groups were randomly selected from dead hamsters in the normal antibody group and normal saline group. In both pcAb (8 mg/kg) groups, there were several negative samples, and the leptospires burden of the other samples was low, including the dead hamster infected with 56606. Therefore, for both the 56601 and the 56606 infection experiments, most of the leptospires in the 3 primary organs were almost completely removed by IgG-pcAb (Fig 5).

## Discussion

First, this study reports the efficacy of a rabbit polyclonal antibody against a homologous and heterologous serotype Leptospira infection in hamster models by assessing the survival rate and leptospiral load in primary organs. The pcAb treatment significantly improved survival in a dose-dependent manner when used to treat 56601 infection. In particular, the survival rate of the highest-dose (16mg/kg) and the second highest-dose (mg/kg) group was 100%. Therefore, a reference dose of IgG-pcAb was provided for treating human or dog leptospirosis. Regardless of whether there was a 56601 infection or a 56606 infection, the pathological injury of organs (kidney, liver and lung) was alleviated, and leptospires in the kidney, liver and lung were almost completely removed compared to the controls. A monoclonal antibody from Balb/c mice or a rabbit polyclonal antibody protected animals from homologous lethal leptospirosis [26,27]. We conclude that the IgG-pcAb derived from rabbits immunized with 56601 can protect hamsters against 56606 infection, a heterologous serotype leptospirosis, but not completely. This provides the possibility of studying of multi-serotype antibodies. In addition, B. H. Jost [26] used a monoclonal antibody 1 hour before challenging with homologous Leptospira, and Toshiyuki Masuzawa[27] administered the antibody 38 days before challenge with homologous Leptospira. We did not treat hamsters before the challenge, but after 48 hours of the challenge, we started treatment. And in the second experiment, combination (pcAb plus antibiotic) after occurrence of the first serious sickness mouse improved survivals compared to pcAb group or antibiotic group.

James E. Moon and Zhang W reported that imipenem and ertapenem induced diarrhea in nearly all hamsters after treatment with these drugs [12,13]. IgG purified from pcAb by caprylic acid ammonium sulfate precipitation method was used for the treatment, as most of extra serum components that may inducing side effects were removed before immunization, it was safer. To assess whether IgG-pcAb was harmful to healthy hamster, a group (n = 10) of healthy hamsters were injected with IgG-pcAb (16 mg/kg). After 21 days, none of hamsters had any clinical symptoms. The activity and appetite of hamsters were also normal (date not show). However, the excellent efficiency of the antibiotic is beyond doubt. For example, cefepime was reported for use in human leptospirosis [34,35]. Therefore, polyclonal antibody treatment and antibiotic therapy can be used together to treat leptospirosis.

This study demonstrates the efficacy of a rabbit polyclonal antibody measured by survival rate, pathological changes and Leptospira burden. The IgG-pcAb can cure the homologous serotype and heterologous serotype Leptospira lethal infection 48 hours after challenge. We also provide a reference dose of IgG-pcAb for treating human or dog leptospirosis. And combination therapy with antibiotic increased survival in homotype leptospiral infection in the late treatment. These results showed the excellent efficacy of IgG-pcAb, which may be a new option for therapy in the future. However, additional clinical research is needed to identify the efficacy in humans.

## References

[1] Ko AI, Goarant C, Picardeau M (2009) Leptospira: the dawn of the molecular genetics era for an emerging zoonotic pathogen. Nat Rev Microbiol 7: 736–747. 10.1038/nrmicro2208 19756012 PMC3384523

[2] Bharti AR, Nally JE, Ricaldi JN, Matthias MA, Diaz MM, et al (2003) Leptospirosis: a zoonotic disease of global importance. Lancet Infect Dis 3: 757–771. 14652202 10.1016/s1473-3099(03)00830-2

[3] Levett PN (2001) Leptospirosis. Clin Microbiol Rev 14: 296–326. 10.1128/CMR.14.2.296-326.2001 11292640 PMC88975

[4] de Vries SG, Visser BJ, Nagel IM, Goris MG, Hartskeerl RA, et al (2014) Leptospirosis in Sub-Saharan Africa: a systematic review. Int J Infect Dis 28: 47–64. 10.1016/j.ijid.2014.06.013 25197035

[5] Adler B, de la Pena Moctezuma A (2010) Leptospira and leptospirosis. Vet Microbiol 140: 287–296. 10.1016/j.vetmic.2009.03.012 19345023

[6] Lau CL, Smythe LD, Craig SB, Weinstein P (2010) Climate change, flooding, urbanisation and leptospirosis: fuelling the fire? Trans R Soc Trop Med Hyg 104: 631–638. 10.1016/j.trstmh.2010.07.002 20813388

[7] Costa F, Hagan JE, Calcagno J, Kane M, Torgerson P, et al (2015) Global Morbidity and Mortality of Leptospirosis: A Systematic Review. PLoS Negl Trop Dis 9: e0003898 10.1371/journal.pntd.0003898 26379143 PMC4574773

[8] Zaki SR, Shieh WJ (1996) Leptospirosis associated with outbreak of acute febrile illness and pulmonary haemorrhage, Nicaragua, 1995. The Epidemic Working Group at Ministry of Health in Nicaragua. Lancet 347: 535–536. 8596276 10.1016/s0140-6736(96)91167-8

[9] Segura ER, Ganoza CA, Campos K, Ricaldi JN, Torres S, et al (2005) Clinical spectrum of pulmonary involvement in leptospirosis in a region of endemicity, with quantification of leptospiral burden. Clin Infect Dis 40: 343–351. 10.1086/427110 15668855 PMC2366057

[10] Gouveia EL, Metcalfe J, de Carvalho AL, Aires TS, Villasboas-Bisneto JC, et al (2008) Leptospirosis-associated severe pulmonary hemorrhagic syndrome, Salvador, Brazil. Emerg Infect Dis 14: 505–508. 10.3201/eid1403.071064 18325275 PMC2570821

[11] Papa A, Theoharidou D, Antoniadis A (2009) Pulmonary involvement and leptospirosis, Greece. Emerg Infect Dis 15: 834–835. 10.3201/eid1505.080270 19402988 PMC2687020

[12] Moon JE, Ellis MW, Griffith ME, Hawley JS, Rivard RG, et al (2006) Efficacy of macrolides and telithromycin against leptospirosis in a hamster model. Antimicrob Agents Chemother 50: 1989–1992. 10.1128/AAC.01467-05 16723556 PMC1479122

[13] Zhang W, Zhang N, Wang W, Wang F, Gong Y, et al (2014) Efficacy of cefepime, ertapenem and norfloxacin against leptospirosis and for the clearance of pathogens in a hamster model. Microb Pathog 77: 78–83. 10.1016/j.micpath.2014.11.006 25450882

[14] Strohl WR, Knight DM (2009) Discovery and development of biopharmaceuticals: current issues. Curr Opin Biotechnol 20: 668–672. 10.1016/j.copbio.2009.10.012 19896824

[15] Chippaux JP, Goyffon M (1998) Venoms, antivenoms and immunotherapy. Toxicon 36: 823–846. 9663690 10.1016/s0041-0101(97)00160-8

[16] Berry JD, Gaudet RG (2011) Antibodies in infectious diseases: polyclonals, monoclonals and niche biotechnology. N Biotechnol 28: 489–501. 10.1016/j.nbt.2011.03.018 21473942 PMC7185793

[17] Casadevall A, Dadachova E, Pirofski LA (2004) Passive antibody therapy for infectious diseases. Nat Rev Microbiol 2: 695–703. 10.1038/nrmicro974 15372080

[18] Guillaume V, Contamin H, Loth P, Georges-Courbot MC, Lefeuvre A, et al (2004) Nipah virus: vaccination and passive protection studies in a hamster model. J Virol 78: 834–840. 10.1128/JVI.78.2.834-840.2004 14694115 PMC368848

[19] Dye JM, Herbert AS, Kuehne AI, Barth JF, Muhammad MA, et al (2012) Postexposure antibody prophylaxis protects nonhuman primates from filovirus disease. Proc Natl Acad Sci U S A 109: 5034–5039. 10.1073/pnas.1200409109 22411795 PMC3323977

[20] Jahrling PB, Geisbert J, Swearengen JR, Jaax GP, Lewis T, et al (1996) Passive immunization of Ebola virus-infected cynomolgus monkeys with immunoglobulin from hyperimmune horses. Arch Virol Suppl 11: 135–140. 8800795 10.1007/978-3-7091-7482-1_12

[21] Jahrling PB, Geisbert TW, Geisbert JB, Swearengen JR, Bray M, et al (1999) Evaluation of immune globulin and recombinant interferon-alpha2b for treatment of experimental Ebola virus infections. J Infect Dis 179 Suppl 1: S224–234. 9988188 10.1086/514310

[22] Kudoyarova-Zubavichene NM, Sergeyev NN, Chepurnov AA, Netesov SV (1999) Preparation and use of hyperimmune serum for prophylaxis and therapy of Ebola virus infections. J Infect Dis 179 Suppl 1: S218–223. 9988187 10.1086/514294

[23] Borisevich IV, Mikhailov VV, Krasnianskii VP, Gradoboev VN, Lebedinskaia EV, et al (1995) [Development and study of the properties of immunoglobulin against Ebola fever]. Vopr Virusol 40: 270–273. 8686265

[24] Mikhailov VV, Borisevich IV, Chernikova NK, Potryvaeva NV, Krasnianskii VP (1994) [The evaluation in hamadryas baboons of the possibility for the specific prevention of Ebola fever]. Vopr Virusol 39: 82–84. 8017061

[25] Dixit R, Herz J, Dalton R, Booy R (2016) Benefits of using heterologous polyclonal antibodies and potential applications to new and undertreated infectious pathogens. Vaccine 34: 1152–1161. 10.1016/j.vaccine.2016.01.016 26802604 PMC7131169

[26] Jost BH, Adler B, Vinh T, Faine S (1986) A monoclonal antibody reacting with a determinant on leptospiral lipopolysaccharide protects guinea pigs against leptospirosis. J Med Microbiol 22: 269–275. 10.1099/00222615-22-3-269 2430103

[27] Masuzawa T, Suzuki R, Yanagihara Y (1996) Protective activity of rabbit polyclonal anti-idiotype antibody against Leptospira interrogans infection in hamsters. Biol Pharm Bull 19: 613–615. 8860969 10.1248/bpb.19.613

[28] Organization WH (2003) Human leptospirosis: guidance for diagnosis, surveillance and control.

[29] Ye C, Yan W, Xiang H, He H, Yang M, et al (2014) Recombinant antigens rLipL21, rLoa22, rLipL32 and rLigACon4-8 for serological diagnosis of leptospirosis by enzyme-linked immunosorbent assays in dogs. PLoS One 9: e111367 10.1371/journal.pone.0111367 25526513 PMC4272274

[30] McKinney MM, Parkinson A (1987) A simple, non-chromatographic procedure to purify immunoglobulins from serum and ascites fluid. J Immunol Methods 96: 271–278. 3805742 10.1016/0022-1759(87)90324-3

[31] Cao Y, Faisal SM, Yan W, Chang YC, McDonough SP, et al (2011) Evaluation of novel fusion proteins derived from extracellular matrix binding domains of LigB as vaccine candidates against leptospirosis in a hamster model. Vaccine 29: 7379–7386. 10.1016/j.vaccine.2011.07.070 21803087

[32] Rojas P, Monahan AM, Schuller S, Miller IS, Markey BK, et al (2010) Detection and quantification of leptospires in urine of dogs: a maintenance host for the zoonotic disease leptospirosis. Eur J Clin Microbiol Infect Dis 29: 1305–1309. 10.1007/s10096-010-0991-2 20559675

[33] Chagas-Junior AD, da Silva CL, Soares LM, Santos CS, Silva CD, et al (2012) Detection and quantification of Leptospira interrogans in hamster and rat kidney samples: immunofluorescent imprints versus real-time PCR. PLoS One 7: e32712 10.1371/journal.pone.0032712 22393440 PMC3290571

[34] Maroun E, Kushawaha A, El-Charabaty E, Mobarakai N, El-Sayegh S (2011) Fulminant Leptospirosis (Weil's disease) in an urban setting as an overlooked cause of multiorgan failure: a case report. J Med Case Rep 5: 7 10.1186/1752-1947-5-7 21235739 PMC3025967

[35] Masuda K, Uehara Y, Ono H, Furukawa K (2010) [A case of severe leptospirosis infection (Weil's disease) in Tokyo]. Kansenshogaku Zasshi 84: 59–64. 20170016 10.11150/kansenshogakuzasshi.84.59

